# Diacylglycerol kinase-ζ regulates mTORC1 and lipogenic metabolism in cancer cells through SREBP-1

**DOI:** 10.1038/oncsis.2015.22

**Published:** 2015-08-24

**Authors:** P Torres-Ayuso, M Tello-Lafoz, I Mérida, A Ávila-Flores

**Affiliations:** 1 Department of Immunology and Oncology, Centro Nacional de Biotecnología/CSIC, Madrid, Spain

## Abstract

Diacylglycerol kinases (DGKs) transform diacylglycerol (DAG) into phosphatidic acid (PA), balancing the levels of these key metabolic and signaling lipids. We previously showed that PA derived from the DGKζ isoform promotes mammalian target of rapamycin complex 1 (mTORC1) activation. This function might be crucial for the growth and survival of cancer cells, especially for those resistant to the allosteric mTOR inhibitor rapamycin. How this positive function of DGKζ coordinates with DAG metabolism and signaling is unknown. In this study, we used a rapamycin-resistant colon cancer cell line as a model to address the role of DGKζ in tumor cells. We found that DGKζ predominated over other PA sources such as DGKα or phospholipase D to activate mTORC1, and that its activity was a component of the rapamycin-induced feedback loops. We show that the DGKζ DAG-consuming function is central to cell homeostasis, as DAG negatively regulates levels of the lipogenic transcription factor SREBP-1. Our findings suggest a model in which simultaneous regulation of DAG and PA levels by DGKζ is integrated with mTOR function to maintain tumor cell homeostasis; we provide new evidence of the crosstalk between mTOR and lipid metabolism that will be advantageous in the design of drug therapies.

## Introduction

The serine–threonine kinase mammalian target of rapamycin (mTOR) is a master signal integrator of growth factors and nutrient levels that coordinates organized cell growth. Hyperactivation of the mTOR complex 1 (mTORC1) downstream of oncogenic mutations in cancer provides the metabolic reprogramming that allows autonomous proliferation and lipogenic tumor metabolism.^1, 2^ The mTORC1 controls protein synthesis by phosphorylating S6 kinase (S6K) and 4EBP-1,^3^ and governs autophagy and glucose metabolism.^1^ The mTORC1 also controls *de novo* lipid biosynthesis by promoting transcriptional regulation of lipogenic enzymes through the SREBP (sterol-regulatory element-binding protein) transcription factors.^4, 5^ The mature active forms of SREBP-1 and -2 are produced by proteolytic cleavage of inactive precursors, and promote transcription of genes needed for fatty acid and cholesterol biosynthesis.^6^ In some systems, mTORC1 regulates SREBP processing by S6K activation,^5, 7^ although additional mTORC1 effectors might also control SREBP processing and activity.^8, 9, 10^

Because of the central role of mTOR in cancer biology, its inhibitors are targets for the development of anticancer drugs. Many of the genetic alterations responsible for colorectal carcinogenesis act through the mTOR pathway, suggesting that mTOR inhibitors could be effective in preventing colon cancer progression. The therapeutic effectiveness of these inhibitors is nonetheless limited, due mainly to the intricate regulation of mTOR and to negative feedback loops that lead to resistance.^11^ Development and evaluation of mTOR-based anticancer therapies require better characterization of the elements that contribute to mTOR crosstalk through different pathways.

The regulation of mTORC1 by the lipid phosphatidic acid (PA) has been proposed to mediate resistance to pharmacological mTOR inhibition. PA activates mTOR by binding to the FKBP12-rapamycin-binding domain (FRB)^12, 13, 14^ that facilitates allosteric mTOR activation and displacement of the endogenous inhibitor FKBP38.^15^ Rapamycin, a potent mTOR inhibitor,^16^ associates with the immunophilin FBPK12 to form a complex that prevents FRB/PA interaction.^12^ Although effective against renal cancer, rapamycin derivatives have only modest effects and limited success in clinical trials for other solid tumors. In advanced cancers, rapamycin-mediated inhibition of mTORC1 releases feedback loops that activate the mTOR complex 2 (mTORC2) and sustain tumor survival via AKT^17, 18^ and SREBP-1-dependent fatty acid synthesis.^19^ The activity of PA-generating enzymes, predicted to limit rapamycin inhibition,^20^ might also temper the rapamycin-triggered feedback loops responsible for resistance in advanced cancers. PA-generating enzyme expression and/or activity could thus provide a basis for cancer therapies.

Diacylglycerol kinase-ζ (DGKζ) is a DGK family enzyme that, by phosphorylating diacylglycerol (DAG), produces PA.^21^ DGKζ-derived PA promotes mTORC1 activation in several cell systems; it promotes mTORC1-mediated phosphorylation of S6K in response to serum in HEK293 cells^22^ and C2C12 myoblasts,^23^ and activates mTORC1 in response to mechanical stimulation of muscle cells.^23^ By controlling TOR function, the *Drosophila* and *Caenorhabditis elegans* DGKζ homologs *rdgA* and *dgk-5* regulate lifespan and the oxidative stress response.^24^ DGKζ-mediated mTORC1 activation might support cancer growth and responses to mTORC1 inhibitors, which coincides with increased DGKζ expression in colon cancer compared with normal tissue,^25, 26, 27, 28^ and with the observation that colon tumors show marked resistance to mTOR inhibition.^29^

Here we used a rapamycin-resistant colon cancer cell line to examine the DGKζ contribution to mTORC1 activation and rapamycin-elicited responses. We confirm that DGKζ operates upstream of mTORC1, limiting its sensitivity to rapamycin, and we identify rapamycin-mediated DGKζ activation as a feedback regulatory circuit by which DGKζ helps to regulate SREBP-1 levels via DAG restriction. As a result, whereas DGKζ depletion sensitizes cells to rapamycin-dependent AKT activation, it severely impairs SREBP-1 stabilization, limiting cell proliferation. Our results show additional DGKζ contributions to the control of mTORC1 functions and provide evidence that DGKζ is a regulatory node in cancer metabolism.

## Results

### DGKζ promotes mTORC1 activation and cell cycle progression in SW480 cells

**Figure 1:**
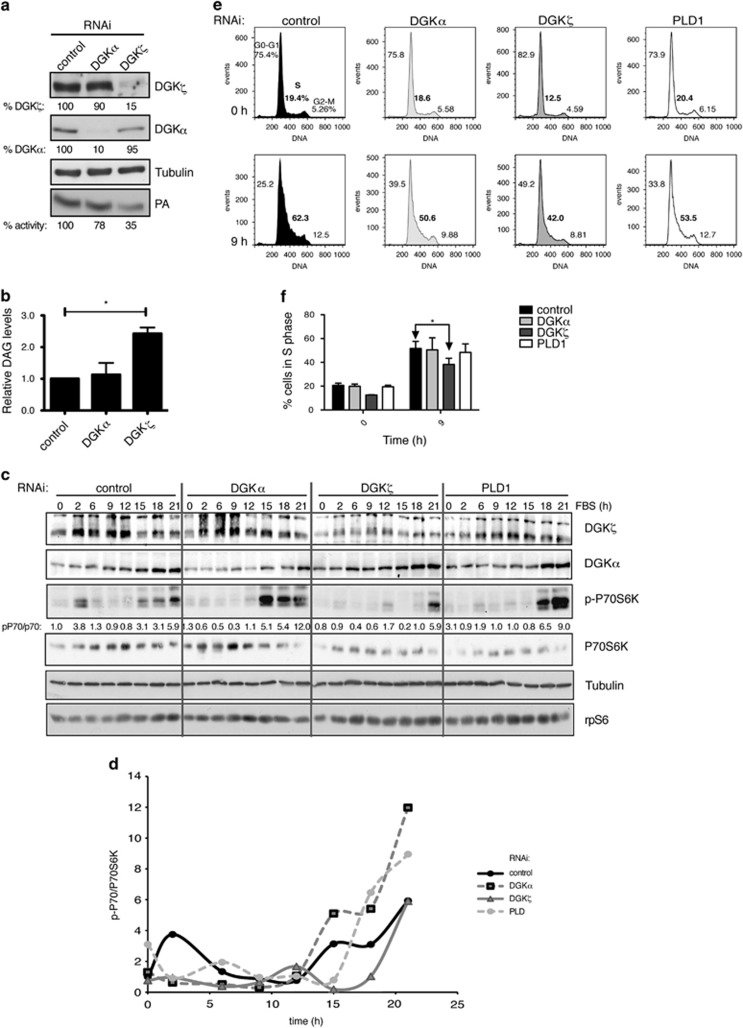
DGKζ activates mTORC1 and promotes G1/S cell cycle progression. (**a**, **b**) SW480 cells were transfected with siRNA, scrambled (control) or specific for DGKα or DGKζ. (**a**) DGK western blot (top) and total DGK activity (bottom). Relative DGK expression and activity are indicated beneath each blot or autoradiograph, as a percentage of control cell expression or activity (100%). Tubulin was used as loading control. (**b**) The same cells were used to determine DAG levels (control cells=1.00) (*n*⩾3). The *t*-test was applied to compare the DAG levels of control and DGKζ-silenced cells. *, significant (*P*<0.05). (**c**–**f**) Cells transfected with siRNA (control or specific for DGKα, DGKζ or PLD1) were serum-starved and cell cycle progression followed after serum re-addition. (**c**) Cell lysates obtained at times indicated were evaluated by western blot for DGK expression and P70S6K phosphorylation levels. P70S6K phosphorylation was normalized to total p70S6K and control cell value at time 0 (1.0). (**d**) Normalized P70S6K phosphorylation is shown for each time. (**e**) Flow cytometry analysis with propidium iodide staining was used to determine the percentage of cells in distinct cell cycle stages. Histograms show times 0 and 9 h of a representative experiment. The percentage of cells in G0–G1, S and G2–M phases is shown in each case. (**f**) Percentage of S-phase cells at 0 and 9 h post serum addition in three experiments. Data were analyzed with analysis of variance (ANOVA); *, significant (*P*<0.05). In (**a**) and (**c**), representative autoradiogram and blots are shown.

The SW480 cell line is derived from a colorectal tumor and has characteristics of highly transformed tumors, including rapamycin resistance.^30^ DGKζ expression was silenced by interfering RNA (RNAi) with a previously validated sequence.^22, 31^ We determined total DGK activity in DGKζ-silenced cells and compared it with that of reducing DGKα, also expressed by this cell line. DGKζ silencing had a greater effect on total DGK activity than DGKα silencing (Figure 1a), in accordance with previous data showing that DGKζ is the main contributor to total DGK activity in these cells.^32^ The main contribution of DGKζ to total DGK activity in SW480 cells correlated with the changes observed in DAG levels after silencing each individual DGK isoform. Total DAG levels were much higher in DGKζ than in DGKα-silenced SW480 cells (Figure 1b).

DGKζ promotes mTORC1 activation in response to serum in renal carcinoma cells^22^ and in C2C12 myoblasts.^23^ We tested the effect of DGKζ silencing by determining phosphorylation of the mTOR substrate p70S6K at various times after serum addition to SW480 cells. As controls, we silenced DGKα and phospholipase D (PLD), another PA-producing enzyme that regulates phosphorylation of the mTORC1 target p70S6K^33^ (Supplementary Figure S1). Serum stimulation promoted an early p70S6K phosphorylation peak, indicating mTOR activation (Figure 1c). A later phosphorylation peak was also observed that probably corresponds to mTORC1 activation at the G2/M checkpoint.^34^ Attenuation of any of these PA-generating enzymes (DGKζ, DGKα or PLD) decreased early p70S6K phosphorylation, but was more pronounced for DGKζ (Figures 1c and d). DGKζ silencing also reduced the later p70S6K phosphorylation peak that was increased by DGKα or PLD silencing. The mTOR activation at the G2/M transition is proposed to allow translation of specific RNAs during mitosis,^34^ although the activation mechanisms are not well understood.

The reduced p70S6K phosphorylation after serum addition correlated with delayed cell entry into S phase (Figure 1e) that was significant only after DGKζ silencing (Figure 1f). These data suggest that after serum stimulation, mTORC1 senses PLD- and DGK-derived PA. The data also hint at a distinct DGKζ contribution to cell-autonomous mTORC1 activation at the G2/M phase.

### DGKζ limits rapamycin cell sensitivity and rapamycin-induced AKT phosphorylation

**Figure 2:**
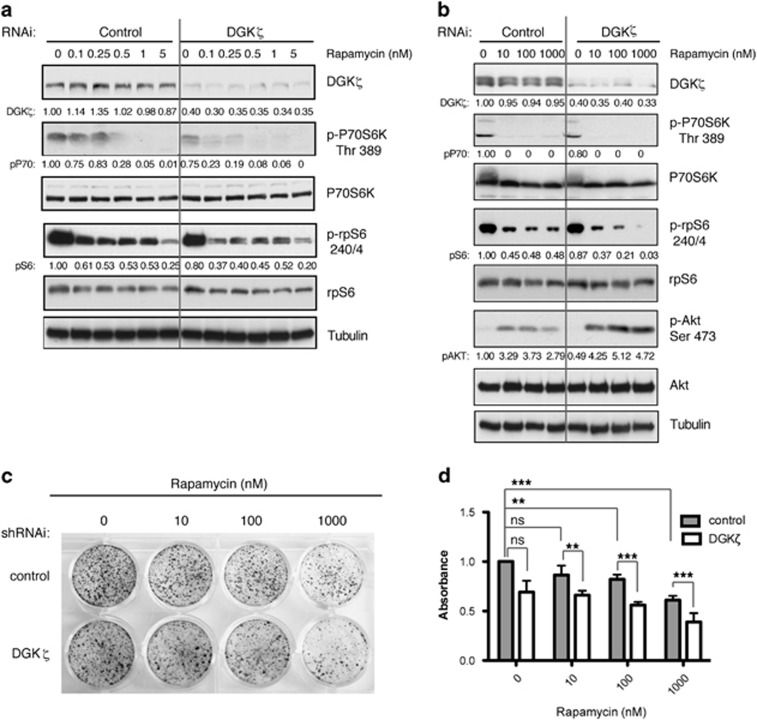
DGKζ silencing modifies rapamycin effects and impairs SW480 cell proliferation. (**a**, **b**) Exponentially growing cells transfected with siRNA control or specific for DGKζ were treated with the indicated rapamycin doses (24 h). Phosphorylation of P70S6K, S6 and AKT were evaluated by western blot in total cell lysates. The expression of the corresponding total protein was determined in separate blots to avoid interference between the distinct antibodies, and tubulin was used as loading control in the same gel to discard changes in protein expression because of rapamycin treatments. Phosphorylation for each protein was normalized to total expression of P70S6K, S6 and AKT, and to control cell values at time 0 (1.0). Representative blots of at least three independent experiments are shown. (**c**) Colony growth formation assays were performed in cells stably transfected with siRNA control or specific for DGKζ and cultured in medium with 10% fetal bovine serum (FBS) and dimethyl sulfoxide (DMSO) or different rapamycin doses. Colony number and size was evaluated by crystal violet staining. (**d**) Mean±s.e.m. of crystal violet absorbance at 620 nm for triplicate samples from one representative experiment (*n*⩾3). Data were analyzed using analysis of variance (ANOVA). ns, non significant; **, significant (*P*<0.01); ***, significant (*P*<0.001).

Rapamycin competes with PA for binding to mTORC1, and the activity of PA-producing enzymes is thought to condition rapamycin effects. To determine whether reducing DGKζ levels increases rapamycin sensitivity of SW480 cells, we examined the effect of low rapamycin doses on p70S6K phosphorylation. Phosphorylation of p70S6K was impaired even at the lowest rapamycin doses (0.1 nM) in cells with reduced DGKζ expression (Figure 2a). Phosphorylation of the ribosomal protein S6, the p70S6K target, was also notably reduced in DGKζ-silenced cells at rapamycin concentrations of <5 nM (Figure 2a), as well as at higher rapamycin doses compared with controls (Figure 2b).

In highly transformed tumor cells, rapamycin-mediated mTORC1 inhibition triggers activation of mTORC2, promoting AKT phosphorylation at Ser473.^35^ Inhibition of p70S6K phosphorylation in rapamycin-treated SW480 cells correlated with increased AKT phosphorylation (Figure 2b). DGKζ depletion enhanced this effect, confirming that limiting DGKζ-mediated mTORC1 activation enhanced rapamycin responses (Figure 2b).

### DGKζ function maintains cancer cell proliferation *in vitro*

Activation of the mTORC2/AKT axis in response to mTORC1 inhibition contributes to the survival of cancer cells in the presence of rapamycin. To test whether, as a result of DGKζ silencing, enhanced AKT phosphorylation contributes to rapamycin resistance in SW480 cells, we studied long-term growth and colony formation in control and DGKζ stably silenced SW480 cells. As predicted, rapamycin reduced colony formation of control cells only at the highest rapamycin doses. DGKζ silencing did not significantly reduce colony formation, but sensitized cells to rapamycin (Figures 2c and d).

### DGKζ is needed to sustain SREBP-1 expression

Our data suggested that although DGKζ expression limits rapamycin-triggered AKT activation, it has additional functions that sustain long-term cancer cell growth. Activation of SREBP-1 cleavage in tumor cells helps to maintain cell-autonomous tumor growth.^4^ The mTORC1 activation of S6K is thought to regulate SREBP-1 processing, giving rise to the active form that promotes transcription, including its own.^5, 7^ SREBP-1 cleavage and transcriptional regulation of its target FASN (fatty acid synthase) are reported to be rapamycin insensitive and AKT regulated in some tumors,^19^ suggesting a role for mTORC2 in the control of lipid metabolism.

**Figure 3:**
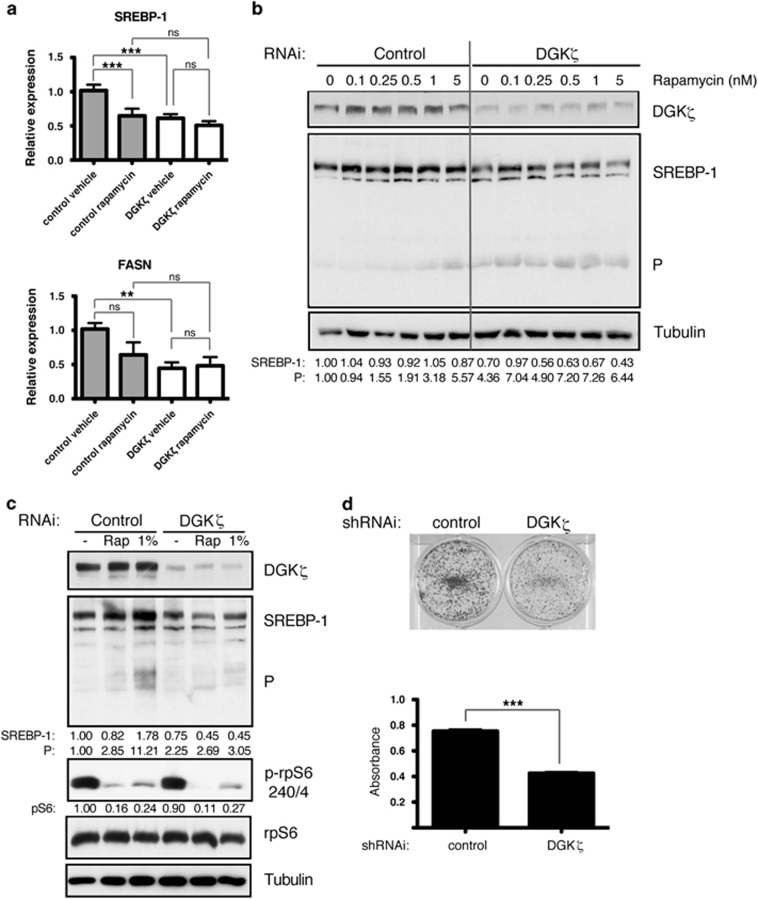
DGKζ controls SREBP-1 levels and transcription activity. (**a**) Cells transfected with siRNA control or specific for DGKζ and in exponential growth were rapamycin treated (100 nM, 24 h). The mRNA levels of *SREBP-1* and *FASN* genes were measured by reverse transcription and real-time quantitative PCR (RT-qPCR). Expression shown is relative to the untreated control. Data were analyzed using analysis of variance (ANOVA). ns, non significant; **, significant (*P*<0.01); ***, significant (*P*<0.001). (**b**) Cells were treated with indicated rapamycin doses (24 h). SREBP-1 levels were evaluated by western blot in total cell lysates. Levels of SREBP-1 and its processed form (P) were normalized to the loading control (tubulin) and control cell values at time 0 (1.0). (**c**) Cells in exponential growth were treated with rapamycin (100 nM) or cultured in medium with 1% fetal bovine serum (FBS; 24 h). SREBP-1 levels and S6 phosphorylation were determined relative to the loading control (tubulin) and total S6, respectively, and control cell values as above. In (**b**) and (**c**), blots are representative of at least three independent experiments. (**d**) Colony growth formation assays of cells cultured in medium with 1% FBS were performed as in Figure 2c. Crystal violet staining (top) and mean±s.e.m. of absorbance at 620 nm is shown (bottom) for triplicate samples from one representative experiment (*n*⩾3). Data were analyzed by *t*-test. ***, significant (*P*<0.001).

We first determined the effect of rapamycin and DGKζ silencing on SREBP-1 transcriptional activity in SW480 cells. DGKζ silencing diminished SREBP-1 and FASN transcription, suggesting reduced SREBP-1 function (Figure 3a). Rapamycin treatment had no significant effect on FASN transcription, although it diminished SREBP-1 transcription, and this concurs with that described for rapamycin-resistant tumors. These data suggest that although DGKζ silencing increased rapamycin-induced AKT activation, it has a negative effect on SREBP-1-mediated transcription.

Reduced SREBP-1 transcription may be the result of impaired proteolytic cleavage of its precursor. We next analyzed whether rapamycin and DGKζ depletion modified SREBP-1 processing. Treatment of SW480 cell with low rapamycin doses increased levels of the processed SREBP-1 form, with no change in the unprocessed form (Figure 3b). This is in agreement with that described above for rapamycin-resistant tumors, in which SREBP-1 processing is AKT mediated. In DGKζ-silenced cells SREBP-1 processing was increased, coinciding with enhanced AKT activation. Our analysis also showed reduced levels of unprocessed SREBP-1 (Figure 3b). These data suggest that although DGKζ silencing increased rapamycin-induced AKT activation, and thus SREBP-1 processing, it impaired precursor levels.

SREBP-1 is closely linked to the capacity of cancer cells to promote lipogenic metabolism; they synthesize the bulk of their lipids from autonomous fatty acids. Reduction of serum levels in SW480 cell culture medium decreased mTORC1 activation to levels similar to those triggered by rapamycin (Figure 3c). SREBP-1 processing increased greatly in these conditions with no diminution in the levels of the nonprocessed form, in agreement with continuous SREBP-1 transcription. As anticipated, DGKζ silencing was not accompanied by maintenance of SREBP-1 levels (Figure 3c). Failure to sustain SREBP-1 processing and transcription correlated with a sharp reduction in long-term growth of DGKζ-silenced cells in low serum conditions (Figure 3d).

### DAG metabolism by DGKζ contributes to sustain SREBP-1 expression

These experiments confirmed the rapamycin insensitivity of SREBP-1 processing in SW480 cells, and suggested that DGKζ is necessary to maintain SREBP-1 precursor levels. SREBP-1 activity might require DGKζ-derived PA or be negatively modulated by a DGKζ-metabolized DAG pool. As we found that DGKζ silencing increased cellular DAG levels, we explored the DAG contribution to the maintenance of SREBP-1 levels.

**Figure 4:**
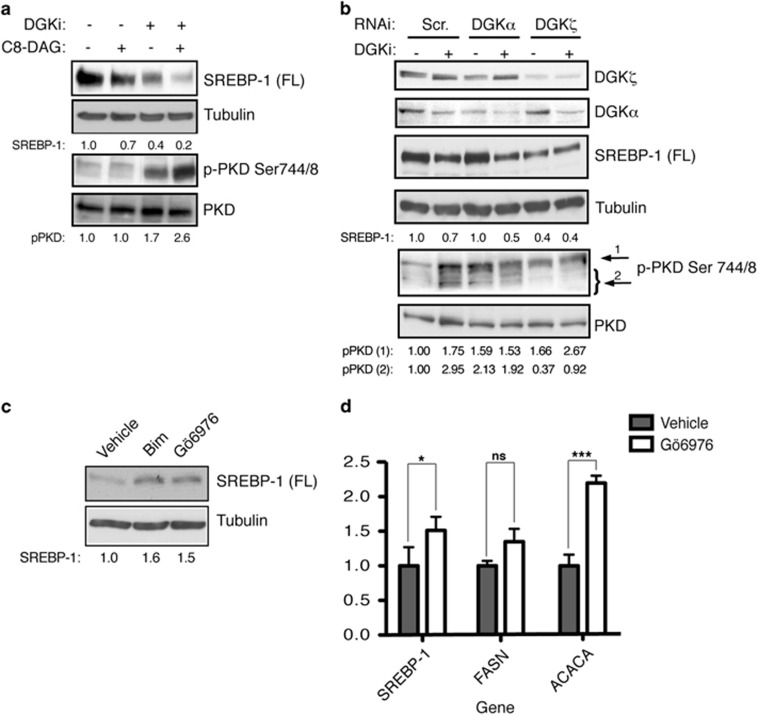
DAG controls SREBP-1 levels. (**a**) SW480 cells were treated (6 h) with C8-DAG (100 μM) or R59949 (30 μM; DGKi), and SREBP-1 levels and PKD phosphorylation analyzed by western blot. (**b**) Western blot analysis of SREBP-1 levels and PKD phosphorylation in cells with silenced DGKα or DGKζ, untreated or treated with R59949 (30 μM). (**c**) Cells were treated with bisindolylmaleimide (Bim) or Gö6976 (100 nM, 16 h), and SREBP-1 levels analyzed by western blot. (**d**) Cells were treated with Gö6976 (100 nM, 16 h) and mRNA levels of indicated genes were measured by qRT-PCR. Data shown for triplicates of a representative experiment (*n*⩾3) relative to untreated controls. Data analyzed using analysis of variance (ANOVA). ns, non significant; **, significant (*P*<0.05); ***, significant (*P*<0.001). In (**a–c**), SREBP-1 levels were normalized to the loading control (tubulin) and control cell values at time 0 (1.0). PKD phosphorylation was normalized to total PKD and control cells. In (**b**), quantification of the two main pPKD bands is shown. In all cases, blots are shown for representative experiments (*n*⩾3).

We determined the effect of DAG on SREBP-1 expression by adding a permeable DAG analog. Protein kinase D (PKD) is activated by direct DAG binding and by protein kinase C (PKC)-dependent phosphorylation;^36^ as PKD phosphorylation is very sensitive to DAG levels, we used it as an indicator of DAG-mediated function. When cells were treated with dioctanoyl (C8)-DAG, western blot analysis showed a modest reduction in SREBP-1 levels and no increase in PKD phosphorylation (Figure 4a). We also examined the consequences of increasing endogenous DAG by treating cells with the DGK inhibitor R59949. Pharmacological DGK inhibition promoted PKD phosphorylation and reduced SREBP-1 levels to a greater extent than C8-DAG. As the reduced C8-DAG effect could be due to DGK-mediated metabolism of the exogenous DAG, we pretreated SW480 cells with the DGK inhibitor before adding the DAG analog. R59949 treatment potentiated the C8-DAG effect, seen as reduced SREBP-1 levels and increased PKD phosphorylation (Figure 4a). These experiments clearly suggest DAG-mediated control of SREBP-1 levels and indicate that DGK-mediated DAG metabolism sustains SREBP-1 activity.

The DGK inhibitor R59949 is reported to block Ca^2+^-sensitive type I DGK, mainly DGKα, more efficiently than other isoforms.^37^ SW480 cells express DGKα but not type I DGKβ and γ.^32^ We compared the effects of DGKζ and DGKα silencing on SREBP-1 levels and PKD phosphorylation with those elicited by pharmacological inhibition of DGK. The sharp reduction in SREBP-1 levels following DGKζ depletion was isoform specific, as DGKα silencing did not alter SREBP-1 expression (Figure 4b). R59949 addition reduced SREBP-1 expression in control and in DGKα-silenced SW480 cells, but not in DGKζ-silenced cells (Figure 4b). R59949 binding to the catalytic domain of DGKα promotes membrane binding of the inactive enzyme favoring dominant negative functions that are not observed when the enzyme is silenced.^32^ The diminished SREBP-1 expression in response to R59949 treatment in control cells could be the result of DGKα inhibition, although the similar effect observed in DGKα-silenced cells suggest the presence of additional R59949-sensitive DGK isoforms and/or a higher sensitivity of DGKζ to the inhibitor upon DGKα silencing. As noted above, inhibitor-treated cells showed increased PKD phosphorylation and slower-migrating bands. The effect of silencing these DGK isoforms on PKD differed from their effect on SREBP-1. DGKα silencing promoted PKD phosphorylation similar to that observed after inhibitor treatment. PKD phosphorylation also increased when DGKζ was silenced that was further potentiated by the inhibitor (Figure 4b). These data correlate with the changes in DAG levels when each DGK isoform was silenced (Figure 1b), and suggest a predominant contribution of DGKζ to DAG clearance. They also demonstrate the DGKα contribution as a negative regulator of specific DAG pools that promote PKD activation.

These experiments suggest that, in SW480 cells, DGKζ-mediated DAG metabolism limits activation of DAG-regulated kinases (PKC and/or PKD), whereas it sustains SREBP-1 function. To determine whether DGKζ regulation of SREBP-1 might be mediated through DAG-activated kinases, we analyzed SREBP-1 levels in cells treated with Bim (a pan-PKC inhibitor) or with Gö6976 (an inhibitor specific for classical PKC and PKD). Both drugs increased SREBP-1 levels, suggesting negative control of SREBP-1-mediated transcription by classical PKC/PKD kinases (Figure 4c). In agreement, we found increased expression of SREBP-1 and its targets in Gö6976-treated cells (Figure 4d). These data indicate that DGKζ controls SREBP-1, at least in part, by modulating a DAG-PKC/PKD effector axis.

### DGKζ is a component of the rapamycin-induced feedback loops

**Figure 5:**
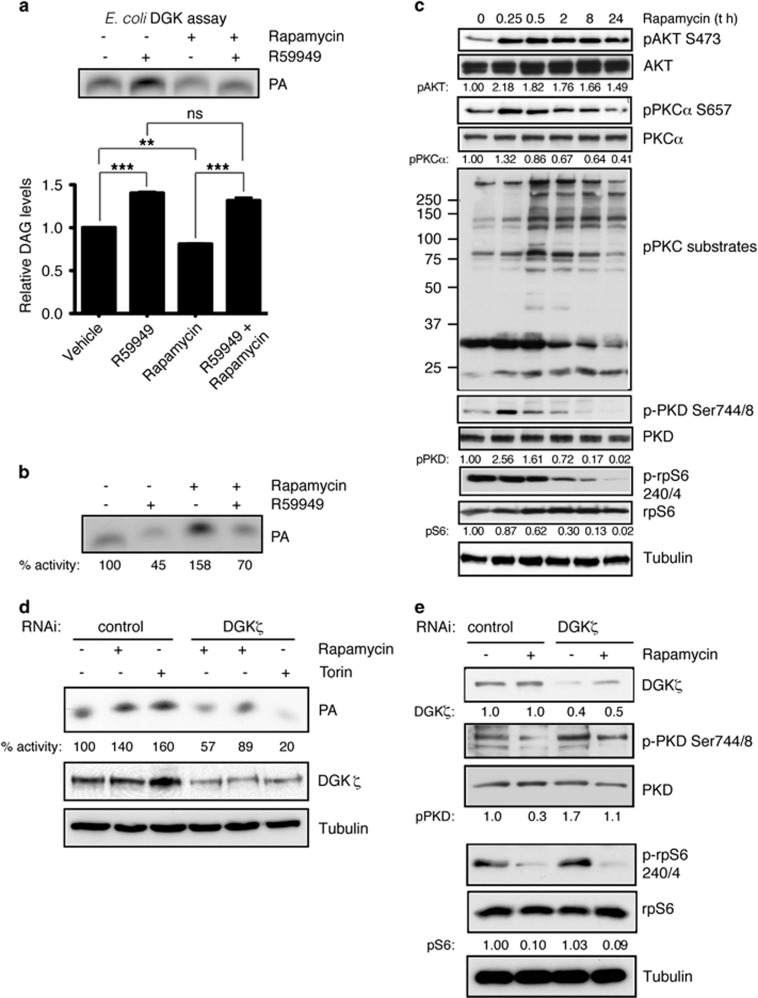
DGKζ participates in the rapamycin-induced feedback loop in SW480 cells. (**a**) Cells were treated with DMSO, R59949, rapamycin or the two inhibitors for 24 h. Total cell DAG was determined in an *in vitro* radioenzyme assay. A representative autoradiogram (top) and mean±s.e.m. of at least three independent experiments are shown (bottom). Data analyzed using analysis of variance (ANOVA). ns, non significant; **, significant (*P*<0.01); ***, significant (*P*<0.001). (**b**) Cells were treated as in (**a**), and DGK activity determined in membrane fractions. Relative DGK activity is indicated beneath a representative autoradiogram of three independent experiments. Activity of vehicle-treated cells=100. (**c**) Cells in exponential growth were rapamycin treated (100 nM) for indicated times, and S6, PKCα, AKT, PKD phosphorylation and PKC activity were tested by western blot in total cell lysates. The expression of the corresponding total protein was determined in separate blots to avoid interference between the distinct antibodies and tubulin was used as loading control in the same gel to discard changes in protein expression because of rapamycin treatments. Phosphorylation for each protein was normalized to total expression of the corresponding protein, and to control cell values at time 0 (1.0). (**d**) Control and DGKζ-silenced cells were treated with rapamycin (100 nM) or torin (250 nM) for 24 h and DGK activity determined. Relative DGK activity is indicated beneath a representative autoradiogram of three independent experiments. Activity of vehicle-treated cells=100. (**e**) PKD and rpS6 phosphorylation was determined in total lysates of cells stably transfected with siRNA control or specific for DGKζ and rapamycin treated (100 nM; 24 h). Phosphorylation was determined as in (**c**), normalized to PKD and S6. Tubulin is shown as loading control. In all cases, blots shown for one representative experiment (*n*⩾3).

DGKζ helps to maintain SREBP-1 levels in rapamycin-treated SW480 cells, suggesting that DGK activation is a consequence of rapamycin-triggered mTORC1 inactivation. Rapamycin treatment decreased DAG levels in SW480 cells, and this effect was reversed by pharmacological DGK inhibition (Figure 5a). Measurement of DGK membrane-associated activity confirmed rapamycin-dependent DGK activation. Rapamycin-mediated DGK activation was observed even in the presence of R59949, correlating with the reduced sensitivity of DGKζ to this inhibitor (Figure 5b).

Our data place DGKζ both up- and downstream of mTORC1, with DGKζ-derived PA facilitating mTORC1 activation and rapamycin-mediated mTORC1 inhibition promoting DGKζ activation. DGKζ associates with and is activated by PKCα in a feedback loop that guarantees precise regulation of PKC activity.^31, 38^ mTORC2 phosphorylates AKT at Ser473 and PKCα at its hydrophobic motif, Ser657.^39^ We reasoned that rapamycin triggering of mTORC2 could activate the PKCα**/**DGKζ axis. Rapamycin treatment of SW480 cells triggered early activation of mTORC2, determined as phosphorylation of AKT and PKCα at their hydrophobic motifs, observed as early as 15 min post rapamycin addition (Figure 5c). AKT remained phosphorylated throughout the experiment, up to 24 h after rapamycin addition. In contrast, PKCα phosphorylation was transient and decreased after 2 h (Figure 5c). In western blot, an antibody that detects PKC-phosphorylated proteins showed rapamycin-triggered PKC activation that decreased at longer treatment times. This phosphorylation was blocked by a pan-PKC inhibitor, confirming PKC activation (not shown). Analysis of PKC-dependent PKD phosphorylation confirmed rapid, transient phosphorylation after rapamycin treatment (Figure 5c). At difference from the total PKC phospho substrate profile, PKD phosphorylation decreased below basal levels at longer treatment times. This transitory effect of rapamycin on PKCα and PKD phosphorylation differs from the dephosphorylation kinetics of the p70S6K target pS6, and correlates well with DGKζ activation.

The previous experiments suggest that rapamycin-mediated mTORC2 activation triggers the mutual regulation of PKCα and DGKζ. To better assess DGKζ regulation downstream of mTORC1 and 2, we compared the effect of rapamycin on DGK activation with that of torin-1 that inhibits both complexes.^40^ In parental SW480 cells, torin-mediated DGK activation was even higher than that found after rapamycin treatment. In contrast to the effect observed for rapamycin, torin treatment did not trigger DGK activation in DGKζ-silenced cells (Figure 5d). These experiments strongly suggest that DGKζ activity is regulated downstream of mTORC2 and the activation of additional DGK isoforms downstream of mTORC1 inhibition in an mTORC2-independent manner.

To further confirm the DGKζ contribution to the regulation of DAG signals downstream of mTORC1 inhibition, we determined the S6 and PKD phosphorylation state in DGKζ-silenced, rapamycin-treated SW40 cells. Long-term rapamycin treatment decreased phosphorylation of both the mTORC1 target S6 and the DAG-effector PKD, as shown above (Figure 5c). The rapamycin effect on PKD phosphorylation was partially lost in DGKζ-silenced cells (Figure 5d). These findings demonstrate that DGKζ limits rapamycin activation of DAG signaling and suggest that it is not the only isoform involved.

### DGKζ limits rapamycin resistance of cancer cells *in vivo*

**Figure 6:**
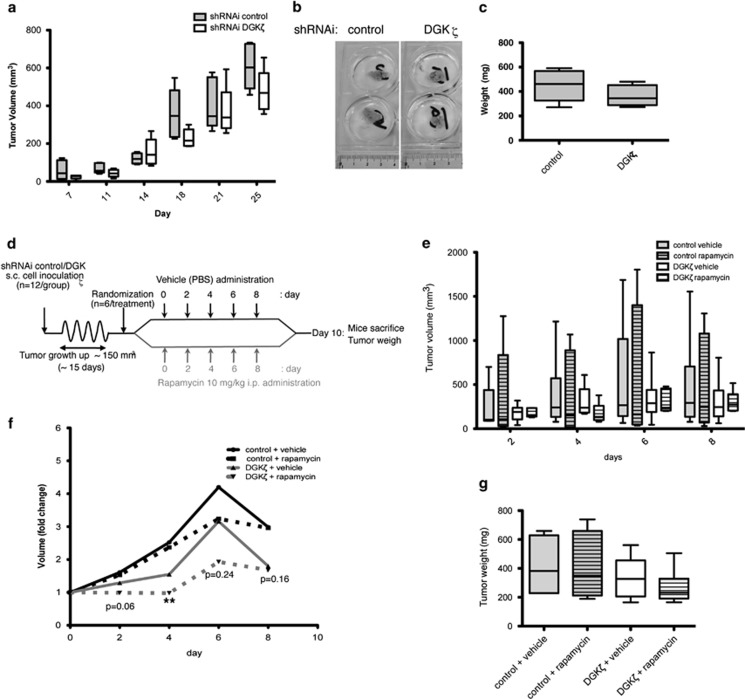
DGKζ silencing enhances the rapamycin effect on cancer cell growth *in vivo*. (**a**) Cells (1.5 × 10^6^) stably transfected with siRNA control or specific for DGKζ were injected subcutaneously (s.c.) into immunocompromised mice and tumor volume recorded every 48 h. Box-and-whiskers plots show evolving tumor volume for each mouse group (*n*=6). (**b**) Tumors removed after killing of the mouse. (**c**) Tumor weight for each group. Data show one representative experiment of three (*n*⩾5 mice/group). (**d**) Test protocol for effects of DGKζ targeting in combination with rapamycin. Cells (10^6^) stably transfected with siRNA control or specific for DGKζ were injected s.c. into immunocompromised mice. When tumors reached ~150 mm^3^, vehicle or rapamycin was administered every 2 days for 8 days. Tumor volume was recorded every 2 days and mice were killed on day 10. Evolving tumor volume for each mouse group (**e**) and mean *x*-fold change (**f**) are shown. After killing, tumors were removed and weighed (**g**). Data show a representative experiment of two performed (*n*⩾6 mice/group). In (**a**, **c**, **e**, **g**) box-and-whisker plots where center line=media; top of box=75th percentile; whiskers=range. In (**f**), **, significant (*P*<0.01), using analysis of variance (ANOVA).

The *in vitro* studies demonstrated that DGKζ helps to sustain SREBP-1 levels in a model in which, through DAG consumption, its activation downstream of mTORC2 is needed to control lipogenic metabolism and long-term cancer cell growth. These properties suggest DGKζ as a potential target for anticancer therapies. We analyzed the DGKζ contribution to cancer growth *in vivo* using stably silenced DGKζ SW480 cells in a xenograft assay model. We injected control or DGKζ-silenced SW480 cells subcutaneously into immunocompromised mice and monitored tumor growth over a 25-day period. Mice that received injections of DGKζ-silenced cells developed tumors that grew more slowly than those derived from control cells (Figure 6a). Most of the excised tumors derived from DGKζ-silenced cells appeared smaller than those from control cells (Figure 6b), although differences in tumor weight at the experiment end point were not statistically significant (Figure 6c). This implies that DGKζ silencing had no marked effect on tumor growth *in vivo*.

To determine whether DGKζ silencing alleviated rapamycin resistance of tumors *in vivo*, we inoculated mice with cells stably expressing control shRNAi or DGKζ shRNAi. When tumors reached 150 mm^3^, mice from each group were subdivided into two random groups for rapamycin treatment (Figure 6d). Given the rapamycin resistance of SW480 cells and our previous *in vitro* data, a large rapamycin dose was administered intraperitoneally every 48 h for 8 days. This dose had no toxic effects on the mice, as inferred from mouse weight (Supplementary Figure S2). DGKζ silencing with rapamycin treatment reduced tumor growth (Figure 6e); this reduction was significant from the outset of treatment, when individual tumor volume was normalized to that measured before treatment (Figure 6f). Analysis of tumors at end point also suggested rapamycin-induced reduction (Figure 6g). These experiments confirmed our *in vitro* observations and suggest that DGKζ depletion sensitizes tumors to rapamycin. DGKζ inhibition might thus be an alternative mechanism to potentiate therapies that target the mTOR axis. In addition, determination of tumor DGKζ levels might be a useful biomarker in the selection of cancer therapy and to predict the effectiveness of mTOR inhibitors.

## Discussion

DGKs control the levels of two essential lipids and are thus predicted to be key components in cancer cell lipid metabolism. Here we extend previous studies of DGKζ-mediated regulation of mTOR by characterizing a functional interrelation of these two proteins in rapamycin-resistant colon cancer cells. Our data provide a link between PA sensing by mTOR and the control of the SREBP-1 transcriptional program by DGKζ-mediated DAG metabolism. We suggest that rapamycin-mediated DGKζ activation in highly malignant cells is a feedback loop that helps to sustain resistance.

We found that DGKζ regulates mTORC1 signaling in the early response to serum addition as well as at late stages of the cell cycle. Other PA-generating enzymes such as DGKα or PLD contribute similarly to early mTORC1 activation, suggesting that a given PA threshold is needed for full mTORC1 activation in response to receptor triggering. Only DGKζ deletion affected the second S6K phosphorylation peak, implying a specific mTORC1 requirement for DGKζ after the receptor-regulated restriction point. The finding of a second S6K phosphorylation peak coincides with reports indicating late mTORC1 activation during the G2/M phase. The mechanisms that operate at this stage differ from those at the G1/S transition, although their nature is unclear. Cyclin-dependent kinase 1 and glycogen synthase 3 are thought to act as mitosis-regulated kinases that phosphorylate the mTORC1 component raptor that activates mTORC1.^34^ mTORC1 activation promotes mRNA translation during mitosis and is thought to be associated to rapamycin resistance.^34^ Nuclear DAG levels increase before mitosis^41^ and activate PKC that acts in concert with CDK1 to mediate lamin B1 phosphorylation and disassembly during mitosis.^42^ How these lipid-mediated events correlate with mitotic mTOR activation and the precise role of DGKζ remain to be determined.

Rapamycin competes with PA for mTORC1 binding that correlates with the enhanced sensitivity of DGKζ-silenced SW480 cells to rapamycin, including activation of mTORC1-controlled negative feedback. These data confirm DGKζ-derived PA regulation of mTOR,^22^ and suggest that rapamycin restriction of PA access to mTOR triggers DGK activation. Our experiments in DGKζ-silenced cells demonstrate activation of additional DGK isoforms by rapamycin. Recent studies demonstrated a central role for DGKα in sustaining oncogenic traits in several types of cancer. Rapamycin resistance leads to Src and Phosphatidylinositol 3-kinase (PI3K) activation,^18^ two signals that mediate DGKα activation. The recent characterization of the DGKα contribution to Src activation in SW480 cells suggests that DGKα activation helps to sustain the feedback circuits released by mTORC1 inhibition.^32^

DGKζ is a substrate of PKCα^38, 43^ that is both an effector and a component of mTORC2 signaling.^44, 45^ Rapamycin short-term kinetics confirmed concomitant phosphorylation of AKT and PKCα at their mTORC2-regulated sites. PKCα phosphorylation of DGKζ triggers its activation, thus terminating DAG-regulated signals.^38^ The sustained AKT phosphorylation contrasted with the transient PKCα phosphorylation and that of its substrates. These results suggest that mTORC2 activation by rapamycin triggers DGKζ that limits DAG-mediated signals. Analysis of torin-treated cells confirmed DGKζ activation downstream of mTORC2, indicating activation of DAG metabolism as component of rapamycin-triggered feedback. This mutual DGKζ and mTOR regulation resembles that of mTOR and PI3K; whereas in normal cells, such axes help to maintain homeostasis, in cancer cells they mediate the cell rewiring that contributes to drug resistance.

The nature of the pathways under the control of mTORC1 feedback loops is understood only incompletely, but these pathways modify cell metabolism and increase survival to sustain cancer cell growth. The mTORC2 is a critical regulator of glycolytic metabolism in cancer, mainly via AKT activation.^46^ SREBP-1 cleavage by AKT-dependent and -independent mechanisms suggest that mTORC2 is also a key controller of lipid metabolism in tumors such as glioblastoma.^19^ We demonstrate that DGKζ silencing regulates SREBP-1 levels while promoting AKT phosphorylation, showing additional controls on SREBP-1. Concomitant addition of DAG and DGK inhibitor also reduced SREBP-1 levels, indicating that DGK-mediated DAG consumption is an AKT-independent mechanism for SREBP-1 regulation downstream of mTOR. R59949 has been reported to block more efficiently the type I DGK isoforms, and from these, α is the only DGK expressed in SW480 cells. The discrepancy between DGKα silencing and the DGK inhibitor effects might be because of the distinct molecular mechanisms that operate in each case, as a reduction in enzyme levels differs from maintaining the enzyme in an inactive conformation. It also probably reflects that R59949 has additional targets, including some unidentified R59949-sensitive DGK isoforms, including DGKζ,

The negative correlation between DAG and SREBP-1 coincides with a report that SREBP-1 activity in liver is negatively regulated by lipin, a PA phosphatase whose nuclear localization is governed by mTORC1-induced phosphorylation.^47^ Specific loss of lipin phosphatase activity in adipocytes decreases DAG, with increased PA and mTORC1 activation.^48^ Our findings support these two studies and suggest a similar DGKζ contribution to the regulation of the mTOR/SREBP axis via modulation of the PA/DAG equilibrium. The mechanism by which increased DAG levels impair SREBP-1 is not clear. Pharmacological classical PKC and/or PKD-1 inhibitors promoted SREBP-1-mediated transcription, strongly suggesting the involvement of these DAG-regulated kinases in control of SREBP-1. DAG might also have a direct effect by altering membrane lipid composition and/or transport.

The activation of DGK lipid kinases in response to mTORC1 inhibition indicates additional feedback mechanisms that restore cell homeostasis. DGKζ activation downstream of mTORC2 in rapamycin-resistant tumors helps to maintain the DAG/PA balance necessary for cell survival. DGKζ reduction potentiates the growth inhibitory effect of rapamycin. In tumors, DGKζ levels could thus control the magnitude of mTORC1-mediated feedback triggered by rapamycin analogs as well as by second-generation mTOR inhibitors. These inhibitors target the mTOR catalytic site and block the mTORC2 complex, preventing AKT activation. They appear to have a biphasic effect on AKT, particularly at Thr308,^49^ but might also target PI3K and trigger feedback mechanisms involving tyrosine signaling.^11, 32^ Determination of tumor DGKζ levels might be a useful marker for therapy assignment and could improve interpretation of therapeutic effectiveness. Our results indicating that cancer cells require DGKζ to maintain lipogenic functions suggest new strategies to better cope with drug resistance in cancer.

## Materials and methods

### Cell culture

The SW480 colon cancer cell line was purchased from the American Type Culture Collection (Manassas, VA, USA), cultured in Dulbecco's modified Eagle's medium (Lonza, Verviers, Belgium) with 10% fetal bovine serum (GBi Genycell Biotech, Granada, Spain) and 2 mM L-Gln (Gibco, Nuaillé, France) and maintained at 37 °C and 5% CO_2_. Cell line identity was confirmed throughout culture by STR genotyping (Genomics Facility, IIB/CSIC, Madrid, Spain).

### Antibodies and reagents

Antibodies to p-S6K Thr389 (9205), S6K (9202), p-AKT Ser473 (9271), AKT (2920), p-S6 Ser240/4 (2215), S6 (2317), p-PKD Ser744/8 (2054) and p-PKC substrate (2261) were from Cell Signaling (Danvers, MA, USA), anti-PKCα Ser657 from US Biologicals (Swappscott, MA, USA) (P9103-16A), anti-PKCα and PKD from Santa Cruz Biotechnology (Santa Cruz, CA, USA) (sc-208, -935, -935), anti-SREBP-1 from Millipore (Temecula, CA, USA) (04-469), anti-DGKζ from Abcam (Cambridge, UK) (ab105195), anti-DGKα from Abnova (Tapei, Taiwan) (BO1P), anti-tubulin DM1A (T9026) from Sigma-Aldrich (Saint Louis, MO, USA) and anti-phosphotyrosine (4G10) from Upstate (Lake Placid, NY, USA). Horseradish peroxidase-coupled polyclonal goat anti-mouse and -rabbit immunoglobulins were from DakoCytomation (Glostrup, Denmark) (P0447, P0448). DGK inhibitor II (R59949; 266788), bisindolylmaleimide and Gö6976 were from Calbiochem (Darmstadt, Germany), (203291, 36520). Rapamycin was from LC Laboratory (Woburn, MA, USA) (R-5000). Torin was from Tocris (Bristol, UK) (4247). Recombinant DGK from *Escherichia coli* (D3065), PMSF, leupeptin and aprotinin were from Sigma-Aldrich. Adenosine 5′triphosphate [γ^32^P] was from Hartman Analytic (Braunschweig, Germany). All lipids were purchased from Avanti Polar Lipids (Alavaster, AL, USA). Organic reagents were from Merck (Darmstadt, Germany).

### Plasmids, siRNA sequences and transfections

For PLD1 targeting, previously described oligos were used,^50^ and knockdown efficiency was assessed.^51^ For DGKζ targeting, we used the previously validated sequences 5′-CUAUGUGACUGAAGAUCGCATT-3′ and 5′-GGUGAAGA GCUGAUUGAGGTT-3′.^22, 38, 52^ DGKα was silenced with validated sequences;^32^ either a scrambled (Ambion, Austin, TX, USA) or the equivalent mouse sequence was used as control. For transient targeting, distinct siRNAs were transfected in cells using Oligofectamine (Invitrogen, Carlsbad, CA, USA). For stable targeting, sequences that target murine (control) or human DGKζ were cloned in the pSuperRetro vector (Oligoengine, Seattle, WA, USA), and SW480 cells stably expressing shRNAi were obtained by infection with pSuperRetro-cloned sequence-containing retroviruses using standard protocols.

### Western blot analysis

For protein extraction from cultured cells and western blot analyses of extracted proteins, we used the ECL system (Amersham, Buckinghamshire, UK) as previously described.^22, 32^

### mRNA expression analysis by PCR or quantitative PCR

Whole RNA extraction, complementary DNA generation, PCR and quantitative PCR were performed as previously described.^32^ Primers for the genes analyzed were SREBP-1, forward 5′-TCAGCGAGGCGGCTTTGGAGCAG-3′, reverse 5′-GAGTCTGCCTTGATGAAGTG-3′ FASN, forward 5′-GAAACTGCAGGAGCTGTC-3′, reverse 5′-CACGGAGT TGAGCCGCAT-3′ ACACA, forward 5′-GCCTCTTCCTGACAAACGAG-3′, reverse 5′-GACTGCCGAAACATCTCTG-3′ GAPDH, forward 5′-ACAGCCTCAAGATCATCA GCAA-3′, reverse 5′-ATGGCATGGACTGTGGTCATG-3′ actin, forward 5′-GGCACCACACCTTCTACAATG-3′, reverse 5′-GTGGTGGTGAAGCTGTAGCC-3′.

### DGK assays and DAG measurement

DGK activity was measured as previously reported,^32^ using 1,2-dioleoyl (C18:1)-DAG or 1,2 dioctanoyl (C8)-DAG as substrate. For DAG measurement, cell lipids were extracted with a modification of the method of Bligh and Dyer^53^ that uses CHCl_3_/MeOH/HCl 12N (50:100:1 v/v/v) in the first extraction step, and the lipid-containing organic phase was dried in a nitrogen stream. DAG and phospholipid-associated phosphate were measured as previously described.^54^

### Exogenous DAG addition

C8-DAG was nitrogen dried and resuspended in vesicle buffer (1 M Tris-HCl pH 8.0, 150 mM NaCl; 5 mM final concentration). Lipids were sonicated to form micelles (5 min, room temperature) and added to cell cultures (100 μM final concentration). DAG or buffer were added every 90 min throughout the treatment period.

### Colony formation assays

Cells (5 × 10^2^/well) were seeded in 12-well plates; medium was replaced with medium containing fetal bovine serum (10 or 1%) after 48 h. Drugs were added at indicated concentrations every 48 h for 7 days. Colony formation was determined as previously described.^32^

### Mouse studies

For all animal work, we used a protocol approved by the CNB/CSIC Ethical Committee for Animal Experimentation (CEEA-CNB, no. 090004), in conformity with national and EU guidelines. Female BALB/c SCID mice aged 6 to 8 weeks received subcutaneous injections of SW480 cells. Tumor volume was estimated as volume = (*a*^2^ × *b*)/2, where *a* is the tumor width and *b* is tumor length in mm. Pharmacological treatment was begun when tumors reached at least 150 mm^3^ (~15 days). The rapamycin dose, selected based on previously determined criteria,^55^ was administered intraperitoneally (10 mg/kg every 48 h for 10 days) and mouse weight was recorded to rule out toxic effects of the treatment. Mice were killed, tumors removed and weighed.

### Statistical analysis

Data are represented as mean±s.e.m.; statistical analysis was done with GraphPad Prism 5 Software (La Jolla, CA, USA). Student's *t*-test was used to compare two data sets. Welch's correction was applied when the variance was significantly different as analyzed with F-test. When samples did not fit normality as tested with the Kolmogorov–Smirnov test, the Mann–Whitney test was used. When more than two conditions were analyzed, we applied analysis of variance and Bonferroni post-test analysis. In all cases, when the *P*-value was <0.05, differences were considered statistically significant (*P<0.05; **P<0.01; ***P<0.001).
