# Supplementary figures and images for: Diacylglycerol kinase-ζ regulates mTORC1 and lipogenic metabolism in cancer cells through SREBP-1
Source: Oncogenesis. 2015 Aug 24;4(8):e164–. doi: 10.1038/oncsis.2015.22 (PMC4632073; doi:10.1038/oncsis.2015.22)

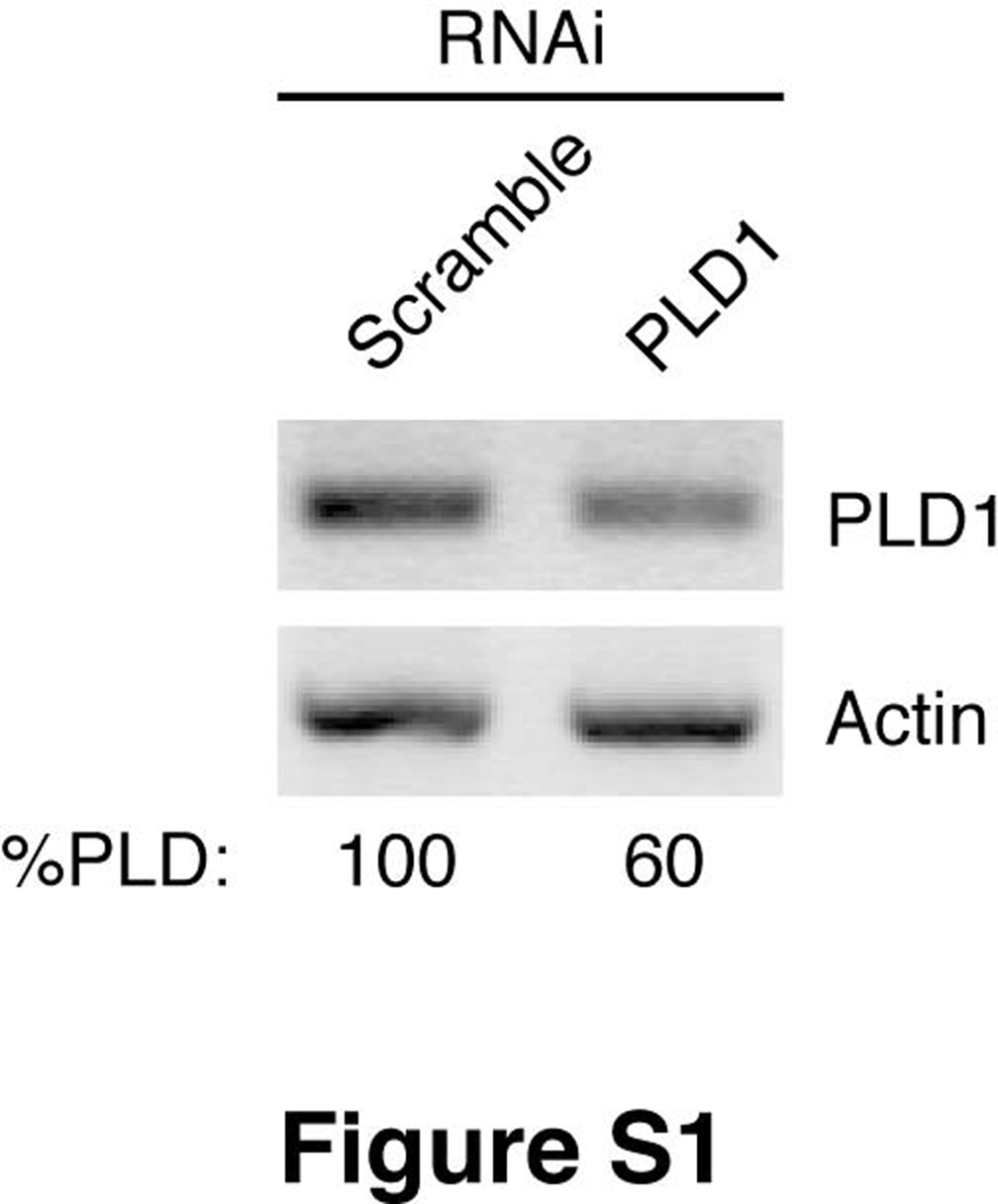

Supplement: Supplementary Figure S1 [file oncsis201522x1.tif]

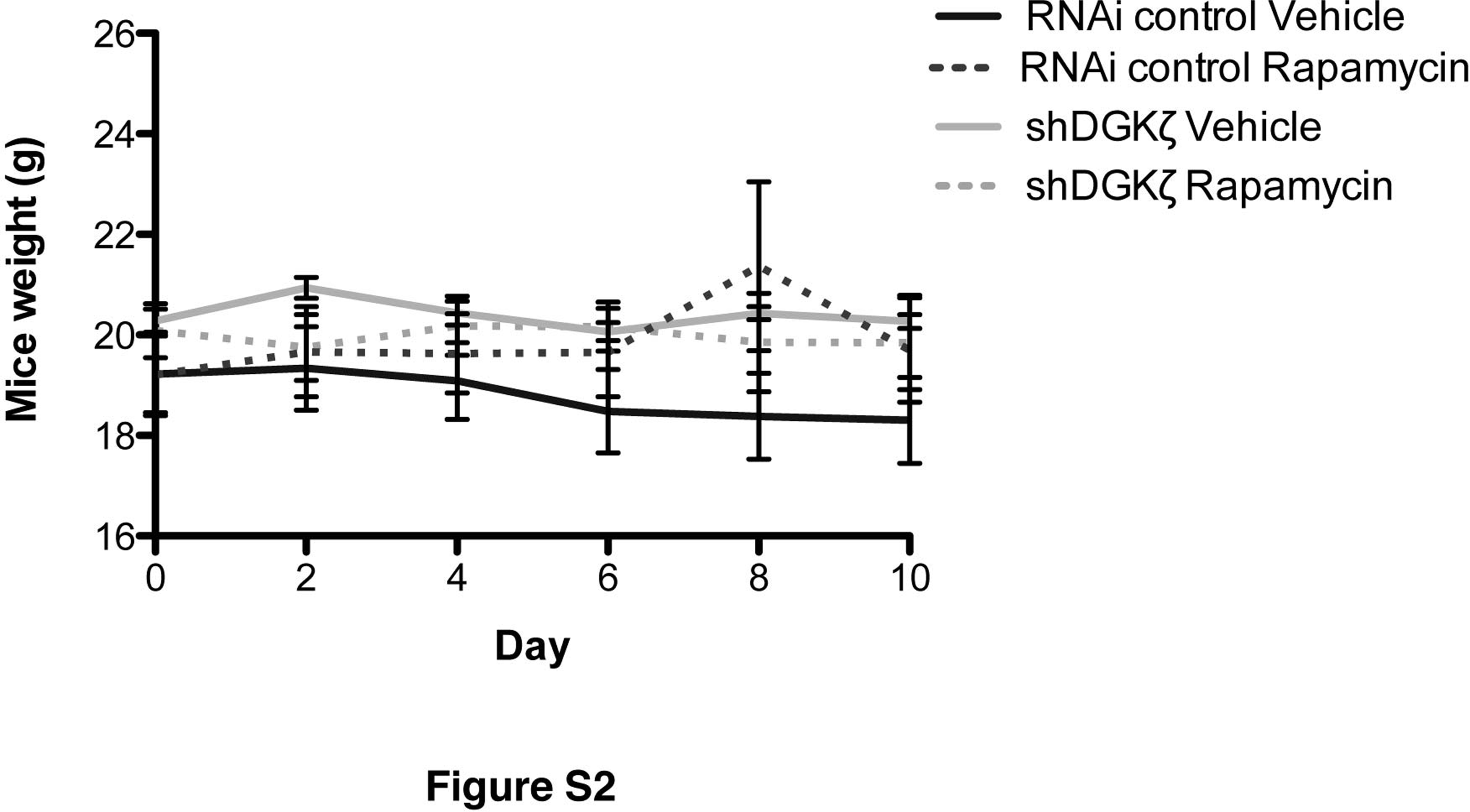

Supplement: Supplementary Figure S2 [file oncsis201522x2.tif]
