# Supplementary material for: Diacylglycerol kinase-ζ regulates mTORC1 and lipogenic metabolism in cancer cells through SREBP-1
Source: Oncogenesis. 2015 Aug 24;4(8):e164–. doi: 10.1038/oncsis.2015.22 (PMC4632073; doi:10.1038/oncsis.2015.22)
Supplement: Supplementary Figure Legends [file oncsis201522x3.doc]

**Supplemental Figue Legends**

**Figure S1. PLD downregulation in SW480 cells.** For transient silencing SW480 cells were transfected with oligofectamine with either a scrambled (Ambion) control sequence or the siRNA against human PLD1 After 96 h post-transfection, cells were lysed and PLD1 levels analyzed by western blot. Actin was used as loading control.

**Figure S4.** **Record of mice weight during Rapamycin treatment.** Mice that received injections of control or DGKζ-silenced SW480 cells were treated or not with Rapamycin and weighted every two days to ensure lack of toxic effects.
